# Towards defining an environmental investment universe within planetary boundaries

**DOI:** 10.1007/s11625-018-0574-1

**Published:** 2018-05-14

**Authors:** Christoph Butz, Jürg Liechti, Julia Bodin, Sarah E. Cornell

**Affiliations:** 1 Thematic Equities, Pictet Asset Management SA, Route des Acacias 60, 1211 Geneva 73, Switzerland; 2 Neosys AG, Privatstrasse 10, 4563 Gerlafingen, Switzerland; 3 Laboratory of Biological Geochemistry, School of Architecture, Civil and Environmental Engineering, École Polytechnique Fédérale de Lausanne, 1015 Lausanne, Switzerland; 4 Stockholm Resilience Centre, Stockholm University, 10691 Stockholm, Sweden

**Keywords:** Planetary boundaries, Sustainable business, Life cycle analysis, Investment, Corporate responsibility, Transdisciplinary dialogue

## Abstract

Science is increasingly able to identify precautionary boundaries for critical Earth system processes, and the business world provides societies with important means for adaptive responses to global environmental risks. In turn, investors provide vital leverage on companies. Here, we report on our transdisciplinary science/business experience in applying the planetary boundaries framework (sensu Rockström et al., Ecol Soc 14, 2009) to define a boundary-compatible investment universe and analyse the environmental compatibility of companies. We translate the planetary boundaries into limits for resource use and emissions per unit of economic value creation, using indicators from the Carnegie Mellon University EIO‑LCA database. The resulting precautionary ‘economic intensities’ can be compared with the current levels of companies’ environmental impact. This necessarily involves simplifying assumptions, for which dialogue between biophysical science, corporate sustainability and investment perspectives is needed. The simplifications mean that our translation is transparent from both biophysical and financial viewpoints, and allow our approach to be responsive to future developments in scientific insights about planetary boundaries. Our approach enables both sub‑industries and individual companies to be screened against the planetary boundaries. Our preliminary application of this screening to the entire background universe of all investable stock‑listed companies gives a selectivity of two orders of magnitude for an investment universe of environmentally attractive stocks. We discuss implications for an expanded role of environmental change science in the development of thematic equity funds.

## Introduction: an investor’s perspective on a sustainability science framework

The planetary boundaries framework (Rockström et al. 2009) was proposed as an approach to tackle urgent sustainability challenges by defining precautionary limits to the human perturbation of key environmental systems. Framework authors Rockström and colleagues argued that these boundaries should be respected absolutely, lest we run the risk of provoking nonlinear and irreversible change from the comparatively stable climatic and ecological conditions of the Holocene, the 12,000-year period in which human civilizations have developed and thrived.

Since the framework was first introduced, researchers from around the world have developed the scientific basis further (discussed in Steffen et al. 2015; Häyhä et al. 2016), and debated how to implement it at national and regional levels (Cole et al. 2014; Dao et al. 2015; Dearing et al. 2014; Nykvist et al. 2013). Yet in order for the planetary boundaries framework to have real influence, its global biophysically expressed boundaries must be operationalized for political and economic actors on all levels, from supranational organisations to countries, and down to industrial sectors and individual companies.

We see investors as playing a crucial part in this debate. Investors provide capital to companies, which means that investment decisions can push the world closer to Earth system thresholds or, alternatively, support the development of intelligent solutions to environmental problems, keeping humanity within its ‘safe operating space’. We therefore argue for an approach that hinges on an iterative dialogue to find common ground between investment management and sustainability science viewpoints. Although the interplay of science, policy and finance is a well-documented feature of the governance of environmental problems (e.g., Dahlmann and Brammer 2013; Pérez-Henriquez 2013; Vatn 2015), there is still very little understanding of the links between the deepening knowledge of the limits to Earth system stability and financial investment and company behaviour (Linnenluecke et al. 2015; Busch et al. 2016).

Here, we describe how we have taken steps to translate the planetary boundaries framework into terms that allow the environmental compatibility of companies to be analysed, for defining a boundary-compatible investment universe. We have experimented with applying our approach in a real-world company analysis, and (without reporting potentially commercially sensitive details of the company analysis itself) we reflect on the choices, challenges and lessons learned from the investment universe identification process.

## Operationalizing the planetary boundaries framework for financial investors

A prime objective of economic activity has been to satisfy the apparently unlimited needs of humans with limited natural resources. In recent decades, the increasing environmental pressure from a growing and more affluent humankind is making the concept of sustainability an important guiding principle of economic activity. In the context of increasing environmental constraints and more stringent regulation, investors play a pivotal role in society’s adaptation to global environmental change. Socially responsible investment (or simply ‘ethical investment’) focuses on more than the conventional measures of financial risk and return, bringing a wider range of social and environmental criteria into financial investment decisions (Sparkes and Cowton 2004; Przychodzen et al. 2016).

Investors should also be interested in the resilience of the longer term economy (Przychodzen et al. 2016). In this context, information provided by the international sustainability science community about globally systemic risks and intergenerational consequences of economic activity is increasingly relevant to investor decision making. In the long term, identifying the companies that are more likely to benefit, rather than being impaired by increasing resource constraints and environmental regulations, will also improve investment decisions and ultimately investment returns (Renneboog et al. 2008).

The scientific community is giving an increasingly clear message that environmental issues cannot be managed individually and separately, but instead need more systemic, integrated analysis and management [e.g., the nexus approach (Hoff 2011; Saundry 2016)]. Asset managers can and should play a more active role in this endeavour by offering their clients intelligent and sustainable investment solutions that deal with multiple environmental issues. Yet further debate is needed about the challenges of translating between biophysical change and financial economy measures so that actions ‘add up’ to global sustainability (e.g., Gimenez and Tachizawa 2012; Hahn and Kuehnen 2013; Bush et al. 2015; Galaz et al. 2016). In this context, the planetary boundaries framework can provide a pragmatic yet credible and scientifically based structure.

Our general approach is to devise measures of economic intensity that are compatible with the environmental limits of our planet. These biophysically acceptable ‘economic intensity boundaries’ (EIB) for resource use and emissions can then be compared to the estimates of actual environmental emissions and resource uses. We used estimates reported in Carnegie Mellon University’s economic input–output life cycle assessment database (EIO-LCA, http://www.eiolca.net), a well-known tool that links over 400 economic activities to their environmental effects, supplemented where needed with environmental and economic information from World Bank Data.^1^

We discuss specific adjustments and assumptions for each of the planetary boundary processes in the following section. We encountered several challenges in this process that serve as caveats as well as pointers to new areas for global change research and for science-business dialogue:

- The planetary boundaries framework specifies a desirable state for nine critical biophysical aspects of the Earth system, but it is not explicit about the path to reach this state. Many social and economic drivers combine to drive the world along its trajectory, whether towards sustainability or a situation of rapidly rising global environmental risks. We have focused on selecting measures of impact that broadly capture company or sub-industry responsibility for pathways to the ‘safe operating space’, rather than attempting a detailed attribution of each biophysical issue among its many possible human-driven causes.
- Planetary and industry processes operate on different time and spatial scales. In several cases, we have selected indicators of short-term, local effects for planetary boundaries that have long-term, globally systemic consequences, rather than attempting to partition Earth’s complex biophysical dynamics into scales that relate directly to corporate decision contexts. This approach embeds a tacit assumption that incremental improvements are enough to avert undesirable shifts in Earth system feedbacks (Jarvis et al. 2012; Ciais et al. 2013). In a sense, this might be a ‘sticking plaster’ approach rather than a treatment of the underlying malaise, but it results in clear signals that companies can actually respond to, and has the obvious benefit of tackling acute environmental problems that would also affect a company’s day-to-day operations.
- There is often no simple correspondence between the ‘control variables’ for Earth system processes selected in the planetary boundaries framework and the measures currently used for corporate environmental performance. Simplifying assumptions had to be made about the causal relationships between EIO-LCA indicators and planetary boundaries processes, to use available information resources in a way that enables businesses to internalize environmental effects now. We opted to include all the most relevant indicators for each boundary, to give the widest scope for leverage on company behaviour. These indicators can then be weighted if necessary to give workable outputs at the stage of sub-industry or company selection. Furthermore, no global input–output model exists to calculate environmental emissions for industry sectors, so the ‘global’ economic intensities calculated from the EIO-LCA database sector data are actually extrapolations from the US economy (the world’s biggest and most interconnected) to the global economy.
- While some of our measures are set at arbitrary levels (albeit in the direction of a desired change), this is in line with the precautionary approach espoused by the planetary boundaries framework (Rockström et al. 2009; Steffen et al. 2015), and can be viewed both as part of adaptive governance and a sustainability learning process (Underdal 2010; Hardt 2013; de Siqueira 2017; Berkes 2017). The vital feature is that, even if arbitrarily set, limits should be traceable and transparent from both a scientific and investor viewpoint.

We do not provide specific company finance analysis data here, only partly because of the obvious commercial and risk implications. More fundamentally, our intention is to demonstrate and explore transdisciplinary issues linking finance, global environmental change and sustainability.

## Translating biophysical control variables into economic intensity measures

In this section, we outline how we translated the planetary boundaries into limits for resource use and emissions per unit of economic value creation, our suggested economic intensity boundaries. Table 1 summarises our assessment of the economic intensity of the planetary boundaries and gives the current value of their respective control variables.

**Table 1:** Translation of biophysical control variables and planetary boundaries to economic intensity measures and investment boundaries

	Biophysical boundary level (Rockström et al. 2009; Steffen et al. 2015)	Current level of control variable (Steffen et al. 2015)	Suggested economic intensity boundary (units per million US$)	Current economic intensity (units per million US$)
Climate change	350 ppm CO_2_	~ 400 ppm CO_2_	188.5 t CO_2_e	639 t CO_2_e
Ocean acidification	Ω^a^ = 2.75	Ω = 2.9	0.0370 kmol H_3_O	0.0282 kmol H_3_O
Stratospheric ozone depletion	276 Dobson Units	283 Dobson Units	2.48 kg CFC-11e	1.05 kg CFC-11e
Biogeochemical flows				
Nitrogen	62 Tg N year^− 1^	140 Tg N year^− 1^		
Phosphorus	11 Tg P year^− 1^	9 Tg P year^− 1^	161 kg Ne^b^	233 kg Ne
Freshwater use	4000 km^3^ year^− 1^	2,600 km^3^ year^− 1^	52,915 m^3^	29,106 m^3^
Land system change	75% original forest cover	62% original forest cover	0.033 kha	0.039 kha
Biosphere integrity—biodiversity loss	10 E/MSY^c^	100 to1000 E/MSY	1.3 × 10^− 7^ E/MSY	17 × 10^− 7^ E/MSY
Atmospheric aerosol loading	NA	NA	3000 n-kg_*AE*_^d^	1000 n-kg_*AE*_
Novel entities—chemical pollution	NA	NA	3000 n-kg_*CP*_	1000 n-kg_*CP*_

Current economic intensities were calculated as the weighted mean of the corresponding values of the 428 US economy sectors listed in EIO-LCA

^a^Ω is the aragonite saturation state

^b^Ne is the ‘nitrogen equivalent’, see “Biogeochemical flows” for explanation

^c^E/MSY is the extinctions per million species per year

^d^n-kg is the virtual emissions unit, for aerosol (AE) and chemical pollution (CP), see “Atmospheric aerosol loading” and “Chemical pollution and environmental release of novel entities” for explanation, respectively

### Climate change

Two values have been proposed (Rockström et al. 2009; Steffen et al. 2015) for the climate change boundary: an atmospheric concentration of 350 ppm CO_2_, and a maximum increase in global radiative forcing of 1 W m^2^. The current CO_2_ concentration is approximately 400 ppm^2^, transgressing the boundary.

A difficulty in operationalizing this boundary is that it is focused on an end state that is impossible for businesses to measure and monitor routinely. Life cycle analysis of products and processes tracks greenhouse gas emissions, not atmospheric concentration. Anthropogenic greenhouse gas emissions (mainly carbon dioxide, methane and nitrous oxide) are extremely likely to be the dominant cause of the observed global warming since the mid-twentieth century, and there is strong scientific consensus about the threat posed by unabated climate change (IPCC 2014). Emissions are generally expressed in CO_2_ equivalents (CO_2_e), an estimate of how many units of CO_2_ would exert the same greenhouse effect as one unit of the gas in question. For example, the impact on climate change of one unit of methane (CH_4_) is roughly 30 times greater than that caused by one unit of CO_2_ over a 100-year period (Myhre et al. 2013). It is tricky to link greenhouse gas emission values to a desired atmospheric CO_2_ concentration level, as concentration does not vary linearly with emissions, given the complexity of the climate system and its various delay and buffer effects (Friedlingstein et al. 2012).

We have therefore made a pragmatic simplification, in line with current policy goals to keep the global climate within 2 °C of warming (UNFCCC 2015). Keeping climate change below a given temperature level requires limiting cumulative global CO_2_ emissions. We use an allowable emissions level of 14.25 billion tonnes CO_2_e per year, corresponding to an emissions limit of 2 tonnes CO_2_e per person per year at current population levels (Schulz et al. 2008; Edenhofer et al. 2014). This limit corresponds to just one-third of the current global average per capita emissions of 6.2 tonnes CO_2_e (World Bank data for 2011, the latest available, http://databank.worldbank.org/data). By dividing this global sustainable emissions figure by global GDP of 75.6 trillion US$ (World Bank data for 2013, http://databank.worldbank.org/data), we derive a planetary boundary-compatible carbon intensity of 188.5 tonnes CO_2_e per million US$:

$${\text{EIB}}~\left[ {{\text{global}}\;{\text{warming}}} \right]=\frac{{{\text{Per}}\;{\text{capita}}\;{\text{sustainable}}\;{\text{emissions}} \times {\text{World}}\;{\text{population}}}}{{{\text{World}}\;{\text{Gross}}\;{\text{Domestic}}\;{\text{Product}}}}$$

The current median carbon economic intensity stands at 639 tonnes CO_2_e per million US$, according to EIO-LCA. Economic intensity would have to come down by 70% to be compatible with the suggested planetary boundary.

A challenge we face in taking this pragmatic approach is that the EIB is sensitive to changes in global GDP. With the planetary boundary as a fixed level for sustainable emissions, a rising GDP would mean a lowered EIB value year on year. However, for climate change, the ‘overshoot’ of the current economic intensity over the boundary-compatible intensity is very large relative to the rate of change of the EIB, and despite a slowdown in recent years, global emissions of CO_2_e are still continuing to rise (Le Quéré et al. 2016). A progressively more stringent EIB could thus be seen as a precautionary approach.

### Ocean acidification

Ocean acidification is the drop in seawater pH caused when acidifying H_3_O^+^ ions form as part of the reaction of atmospheric CO_2_ (and other acid agents) with seawater. This acidification shifts the chemical equilibrium of the marine carbonate reaction, making carbonate ions less abundant and the formation of carbonate minerals more difficult. These minerals are the molecular building block of the skeletons and shells of many marine organisms, so ocean acidification can affect economically important shellfisheries and fisheries (Cooley and Doney 2009; Gattuso et al. 2015), and also the global carbon cycle and climate feedback processes (Hofmann and Schellnhuber 2009).

The planetary boundary (Rockström et al. 2009) is defined in terms of the marine saturation level, Ω, of aragonite, a form of calcium carbonate. The boundary is set at a minimum Ω value of 2.75, or 80% of the pre-industrial level of 3.44, and the current Ω is approximately 2.9 (Feely et al. 2009).

This definition is not readily operationalizable by businesses. It is specified in terms of a target equilibrium value of dissolved ions in seawater, not an absolute concentration, whereas what is needed is an assessment of the emissions level of CO_2_ or other acidifying substances that would allow us to stay within the proposed boundary. To make the boundary operational, we have taken a multi-stage approach linking acidification in the ocean to the emission of acidifying substances.

We consider four indicators of acidifying substances, CO_2_, NO_2_, SO_2_ and NH_3_, for which the EIO-LCA database provides approximate values of the global emissions. Industry is responsible for a large proportion of incineration and combustion processes which do not emit CO_2_ alone, but also large amounts of other gases that act as acids when they come into contact with water, especially the nitric and sulfur oxides NO_2_ and SO_2_ which also contribute to H_3_O^+^ formation. The acids formed when these gases dissolve, HNO_3_ and H_2_SO_3_, are much stronger than the H_2_CO_3_ formed when CO_2_ dissolves, meaning that they form more H_3_O^+^ molecules per mole of dissolved substance. On the other hand, a lower total quantity of them is emitted and they are more easily washed out of the atmosphere by rainfall than CO_2_. This means that they tend to be washed out on land (close to their emission site), acidifying soils and inland waters. We nevertheless include these substances in the ocean acidification boundary using an estimated washout factor as recent papers give evidence that they contribute to alterations in surface water chemistry (Doney et al. 2007; Kim et al. 2014).

Using the known acid dissociation constants of the acidifying substances, the EIO-LCA emission intensities of these substances and some assumptions on the probability for outwashing of these substances at sea, we have defined a total ocean acidification economic intensity of 0.0282 kmol H_3_O^+^ per million US$. This corresponds to 2.1 million kmol H_3_O^+^ per year, dominated by CO_2_ and NO_2_.

To link the economic intensity of acidifying emissions to the planetary boundary, we used Guinotte and Fabry’s estimate (Guinotte and Fabry 2008) of an aragonite saturation of 2.29 for a doubling of the atmospheric CO_2_ concentration. Using known atmospheric CO_2_ concentration and aragonite saturation for pre-industrial conditions, present day and this projected state, we fit a logarithmic relationship ([CO_2_] = − 687.4 × ln (Ω_arag_) + 1130.3; *R*^2^ = 0.9999) and estimated that the Rockström et al. (2009) aragonite saturation boundary of 2.75 corresponds to an atmospheric CO_2_ concentration of 435 ppm. For CO_2_, then, the current acidification state is at about 76% of the boundary value.

In the absence of more detailed biogeochemical information, we have assumed that this acidification state margin applies across the suite of acidifying substances at their current levels (2.1 kmol H_3_O^+^ per year, above), giving us a boundary level of acidifying emissions of 2.8 million kmol H_3_O^+^ per year.

If we divide this value by the current GDP, the economic planetary boundary-respecting ocean acidification economic intensity is 0.0370 kmol H_3_O^+^ per million US$ of turnover.

$${\text{EIB}}\left[ {{\text{ocean}}\;{\text{acidification}}} \right]=\frac{{{\text{Current}}\;{\text{emissions}}}}{{{\text{Acidification}}\;{\text{state}} \times {\text{World}}\;{\text{GDP}}}}$$

### Stratospheric ozone depletion

Stratospheric ozone plays a crucial role in filtering out life-threatening ultraviolet radiation from the Sun. In the early 1980s, it became evident that high atmospheric concentrations of synthetic ozone-depleting substances (ODS) had crossed a threshold and were triggering chemical chain-reactions on polar stratospheric clouds over the Antarctic, leading to what became widely known as the Antarctic ‘ozone hole’.

Fortunately, the ozone hole is not just a textbook example for an unexpected and sudden transgression of a planetary boundary, but it is also a prominent example of a concerted and speedy human response (Andersen et al. 2013). Thanks to the 1987 Montreal Protocol that banned the most harmful ODS, global ODS emissions have been decreasing since 1990. In the analysis of Rockström et al. (2009) and Steffen et al. (2015), this boundary is not transgressed anymore. Nevertheless, the Antarctic ozone hole is expected to continue to exist for some decades, and Arctic ozone losses may also continue for the next decade or two, so we have included it in our derivation of economic boundaries.

The proposed planetary boundary for stratospheric ozone levels is 276 Dobson Units (DU), which corresponds to a maximum allowable depletion of 5% below the pre-industrial ozone levels of 290 DU. Present levels are at 283 DU.

It is difficult to match actual ODS emissions with a given stratospheric ozone level, due to the complexity of chemical reactions and the time lags involved. For instance, it took 15 years after the Montreal Protocol for the stabilising effects on ozone levels to become evident (NOAA et al. 2011).

Our approach is to compare values for observed ozone levels and ODS emissions over time. The 1980 emission levels of ODS of 6.6 billion tonnes of CO_2_e per year were low enough to result in a steady state by 1995, when a stabilisation of concentrations in the ozone layer was observed (IPCC and UNEP 2005). When annual ODS emissions fell further to 2.8 billion tonnes CO_2_e, observed ODS concentrations in the atmosphere began to decrease, allowing the ozone layer to recover. We have therefore taken the 1980 level of emissions as a planetary boundary not to be transgressed. The current level of global emissions, at about 40% of the emission levels reached in the 1980s, seems sustainable and with continued international controls, there is a margin of safety.

We have then applied the ratio between today’s (lower) emissions levels and the estimated ‘steady state’ emission levels of the 1980s to the current industrial ozone depletion intensity given in the EIO-LCA database. This gives a boundary-compatible economic ozone depletion intensity of 2.48 kg CFC-11 equivalent per million US$. The economy’s current average ozone depletion intensity, according to EIO-LCA, stands at 1.05 kg CFC-11 equivalent per million US$.

$${\text{EIB}}\left[ {{\text{ozone}}\;{\text{depletion}}} \right]=\frac{{{\text{Boundary}}\;{\text{ODS}}\;{\text{emission}}\;{\text{level}}~}}{{{\text{Current}}\;{\text{ODS}}\;{\text{emission}}\;{\text{level}}~}} \times {\text{Current}}\;{\text{ODS}}\;{\text{emission}}\;{\text{intensity}}$$

### Biogeochemical flows

The planetary boundary for biogeochemical flows actually consists of two boundaries: phosphorus (P) and nitrogen (N). These elements are macronutrients, and their supply rules the development of organisms on many levels. Human modification of the N and P cycles is profound (Sutton et al. 2013): atmospheric nitrogen gas is ‘fixed’ industrially into reactive forms of N, and phosphate minerals are mined and converted into reactive forms of P. The primary purpose of most of the N and P use is food production, but much reactive N and P eventually ends up in the environment. This ‘open loop’ economy is increasingly recognised as a severe globally systemic problem (Fowler et al. 2013).

Anthropogenic P and N influxes alter the environmental balance of the elements that living organisms use. This can trigger nonlinear shifts in ecological conditions, because some species thrive better than others under different nutrient conditions. The effects of nutrient enrichment have been most obvious in lakes and rivers, which can be pushed over the threshold of eutrophication resulting in undesirable or harmful algal blooms, depletion of oxygen in the water, and the death of aquatic plants and fish. Despite improved fertilizer management and restrictions on the use of phosphorus in household products in parts of the world, there is still a strong input of P and N into watercourses through diffuse sources of pollution (Bouwman et al. 2013).

In Steffen et al. (2015), both the N and P boundaries relate to the problems of eutrophication, which is a category of impact in the EIO-LCA database. Rather than implementing two different boundaries, especially given our inability at this stage to integrate the sub-global boundaries suggested by Steffen et al. (2015), we have devised one global aggregated boundary for eutrophication.

To do this, we translate P into N equivalents (N-e). The CML-IA Characterisation Factors database^3^ provides an equivalence value for eutrophication of 0.42 kg PO_4_^−^ for 1 kg N in freshwater. The boundary of 11 million tonnes P per year from Rockström et al. (2009) corresponds to 80.3 million tonnes N-e per year. Adding this to the N planetary boundary gives a global biogeochemical flows boundary of 142.3 million tonnes N-e per year.

Using the same procedure for the current global N and P flows given in Rockström et al. (2009) and Steffen et al. (2015) gives a total combined anthropogenic N and P influx of 205.7 million tonnes N-e per year. The boundary has been exceeded by a factor of 1.44.

EIO-LCA gives an average current economic eutrophication intensity of 233 kg N-e per million US$ (for air and water). By dividing this value by the ‘transgression factor’ of 1.44, we obtain a boundary-compatible eutrophication intensity (EIB [Eutrophication]) of 161 kg N-e per million US$ of economic value.

$${\text{EIB}}\left[ {{\text{eutrophication}}} \right]=\frac{{{\text{N}}\;{\text{and}}\;{\text{P}}\;{\text{boundary}}}}{{{\text{Total}}\;{\text{N}}\;{\text{and}}\;{\text{P}}\;{\text{influx}}}} \times {\text{Current}}\;{\text{eutrophication}}\;{\text{intensity}}$$

### Freshwater use

Global manipulations of the freshwater cycle have adverse effects on water availability and quality, impacting biodiversity, the functioning of ecosystems, human health and food security. Water use also frequently sparks political tensions, both within countries and across national boundaries. The planetary boundaries framework focuses on the loss of soil moisture (green water) associated with land degradation and deforestation, and the shifts and abstraction of surface water (blue water). In addition, groundwater tables are falling in many parts of the world as aquifers are ‘mined’ at faster rates than they can be replenished.

Rockström et al. (2009) indicate a planetary boundary of ~ 4000 km^3^ per year of consumptive water use, with current use levels estimated at ~ 2600 km^3^ per year (Shiklomanov and Rodda 2003). Steffen et al. (2015) introduced a basin-specific control variable: a withdrawal rate expressed as a percentage of mean monthly river flow. This basin-specific approach is not currently compatible with the EIO-LCA, so we therefore continue to use the globally defined boundary.

Water withdrawal is the most important EIO-LCA indicator for freshwater use, but we have also included the EIO-LCA indicators for toxic releases to surface and underground water into our calculation of the boundary. We consider toxic emissions to water as another form of water use, because water pollution can render water unusable and thus reduce effective water availability. The freshwater-related indicators in EIO-LCA are given in different units: water withdrawal in thousands of gallons (kGal; 1 kGal = 3.7854 m^3^), and pollution in kg of toxic releases to water. For toxic releases, we have assumed a toxic concentration limit of 10 mg per liter (10 ppm) of a given pollutant, so 1 kg of toxic emissions to underground or surface water will render 100 m^3^ of water unusable for consumption.

The ratio of global water withdrawal to water consumption given in Rockström et al. (2009) is 1.53. If we apply this factor to the proposed consumptive use boundary, this takes us to an allowable water withdrawal of 6,154 km^3^ per year. Dividing this figure by the global GDP gets us to a boundary-compatible water withdrawal intensity of 81,408 m^3^ per million US$. (In terms of consumptive use, this corresponds to 52,915 m^3^ per million US$.)

The current average economic intensity of freshwater use derived from EIO-LCA is 29,106 m^3^ per million US$.

$${\text{EIB}}\left[ {{\text{water}}~\;{\text{use}}} \right]=\frac{{{\text{Water}}\;{\text{withdrawal}}}}{{{\text{Water}}\;{\text{consumptive}}\;{\text{use}}}} \times ~\frac{{{\text{Freshwater}}\;{\text{boundary}}}}{{{\text{World}}\;{\text{GDP}}}}$$

### Land system change

Land is the ultimate limited resource: less than one-third of our planet’s area is land, of which only a fraction supports vegetation growth. Forests play a paramount role in safeguarding and stabilising the soil, the water cycle, and the whole climate system. Forests act as carbon sinks, and are among the most biologically diverse habitats on Earth, but forested land has been converted to agricultural use at a rate of nearly 1% per year for the past 50 years (MA 2005). Today’s forest cover accounts for roughly 4 billion hectares (FAO Forestry 2010). Steffen et al. (2015) report that this represents 62% of the pre-industrial forest cover.

The suggested planetary boundary for land conversion to cropland in Rockström et al. (2009) is of no more than 15% of the global ice-free land surface. In Steffen et al. (2015), the focus is on forest preservation, and a regional dimension is introduced, specifying different boundaries for tropical, temperate and boreal forest biomes because of their different functions in the Earth system. For our operationalization, we have taken the weighted average of these three forest biomes, and use a global boundary value of 75% of the pre-industrial forest cover. At this stage, we are unable to account for regional differences in our model. Nevertheless, on an individual company level, it may well be useful to take the regional exposure and impacts into consideration.

Applying this minimum forest preservation rate of 75% gives just under 5 billion hectares of forests to be kept intact if we want to remain within the safe operating space. Globally, the useable land surface is approximately 13 billion hectares, leaving a land area of 8.3 billion hectares that theoretically could be used for human purposes. Dividing this amount by the latest global GDP of 75.6 trillion US$ would result in a boundary-compatible economic intensity of 0.110 thousand hectares per million US$. However, most land take for human use is occurring on forested land (Lambin and Meyfroidt 2011; Lindquist et al. 2012). We apply a tentative 30% probability that further land take would occur on the non-forested land. This reduces the boundary-compatible economic intensity of land use to 0.033 thousand hectares per million US$.

The current average economic intensity of land use derived from EIO-LCA is 0.039 thousand hectares per million US$, thus transgressing the sustainable threshold.

### Biodiversity loss

Biodiversity is the huge gene bank that represents the potential of life on Earth to adapt and respond to changing environmental conditions. It also sustains important life-supporting ecosystem services (Mace et al. 2014; Steffen et al. 2015). Anthropogenic activities have accelerated the extinction rate, which currently stands between 100 and 1000 extinctions per million species-years (E/MSY). Many aspects of biodiversity loss are highly regional, so we aim to include regional aspects in our individual company analysis, but for now we use a global approach for our operationalisation.

Rockström et al. (2009) suggest a boundary of 10 E/MSY. This is already 10 to 100 times the natural rate (Mace et al. 2005). Dividing the number of ‘acceptable’ extinctions by the global GDP, 75.6 trillion US$, gives us a boundary-compatible economic intensity of species loss of 1.32 × 10^7^ E/MSY per million US$.

The EIO-LCA tool does not provide specific indicators for biodiversity loss, so we selected indicators that are relevant for biodiversity impacts (CBD 2014): land-use change, global warming, eutrophication and eco-toxic releases. We have conservatively used the lower reported rate of current species extinction, 100 E/MSY, and distributed those extinctions over the four selected indicators, weighted according to their estimated impacts. We calculated E/MSY per unit value for each impact, and used this as the conversion factor applied to the respective intensities of economic value creation from the EIO-LCA database. This yielded the ‘economic intensity of biodiversity loss’ for each of the indicators.

The sum total of all these intensities is 17.4 × 10^− 7^ E/MSY per million US$ of value creation, greatly in excess of the ‘acceptable’ boundary value of 1.32 × 10^− 7^ E/MSY per million US$. These indicators, our tentative weighting assumptions and the resulting estimates of species loss per indicator are summarised in Table 2.

**Table 2:** Translation of biophysical control variables for biodiversity loss to economic intensity measures and investment boundaries

	EIO-LCA impact indicator			
	Land-use change	Global warming	Eutrophication	Ecotoxic releases
Unit	kha	t CO_2_e	kg N-e	kg 1,4DCBe^a^
Current value	1.6 million	45 billion	205 billion	12.6 billion
E/MSY per unit	3.13 × 10^− 5^	5.55 × 10^− 10^	7.32 × 10^− 11^	7.94 × 10^− 10^
Weighting	50%	25%	15%	10%
Current economic intensity, units per million US$	0.039 (median)	642,000 (median)	232 (air) 0.72 (water)	88 (low) 94 (high)
EI [biodiversity loss], E/MSY per million US$	12.2 × 10^− 7^	3.6 × 10^− 7^	1.7 × 10^− 8^ (air) 5.3 × 10^− 11^ (water)	7 × 10^− 8^ (low) 7.4 × 10^− 8^ (high)

^a^The LCA characterization reference factor DCBe is dichlorobenzene equivalent

### Atmospheric aerosol loading

The planetary boundaries framework (Rockström et al. 2009; Steffen et al. 2015) makes a strong case for including atmospheric aerosol loading among the nine key environmental dimensions, although it does not provide a quantitative boundary. Aerosols are small airborne particles released to the atmosphere by both natural and human activities. They exert a strong influence on the climate system, hydrological cycle, and atmospheric chemical processes. They also have multiple adverse effects on organism health. These different impacts of aerosol loading act on different geographical scales, and the planetary boundaries framework focuses on the Earth system impacts, while EIO-LCA tends to focus on the local air quality and human health impacts. Global mean values are of limited use to address those localised problems. However, local emission controls motivated by human health impacts may provide important leverage for the larger-scale environmental impacts.

For our model, we have tried to establish a first tentative economic intensity boundary for aerosols, combining seven relevant indicators from the EIO-LCA database into one aggregated value for aerosol loading (Table 3). The EIO-LCA aerosol indicators can be broadly classified in two groups: general macro emissions (PM10, PM2.5 and VOC), and emissions with adverse effects on human health (via critical air particulates, smog, cancer and non-cancer). In EIO-LCA, the human-health based impacts are expressed in the widely used impact unit Disability Adjusted Life Years (DALY), so they had to be translated into measures of their relative environmental contribution.

**Table 3:** Translation of biophysical control variables for atmospheric aerosol to economic intensity measures and investment boundaries

EIO-LCA indicator, *i*	Unit (per million US$)	Average value in original unit	Conversion factor	Average value in n-kg_*AE*_
Macro emissions				
PM10	Tonne	2.46	51	125
PM2.5	Tonne	0.7	181	125
VOC	Tonne	1.1	232	250
Human Health criteria				
Critical air particulates	kg PM10e	3451	0.044	150
Smog	kg O_3_e	71,534	2.8 × 10^− 4^	20
Cancer (high)	kg benzene	1331	0.0015	2.1
Cancer (low)	kg benzene	199	0.0015	0.3
Non-cancer (high)	kg toluene	1,963,657	1.6 × 10^− 4^	310
Non-cancer (low)	kg toluene	116,853	1.6 × 10^− 4^	18
Total	n-kg_*AE*_			1000

We converted the seven EIO-LCA indicators into a virtual aerosol unit, n-kg_*AE*_, by first arbitrarily setting a total of 1000 n-kg_*AE*_ per million US$ (Table 3). We calibrated the contribution of the current levels of the EIO-LCA agents to obtain 500 n-kg_*AE*_ for each group. For the first group, we applied weightings according to our own simplifying assumptions about the relative contributions to the aerosol problem (equal weights for PM10 and PM2.5, double the weight for VOC). For the second group, weightings were based on reported DALY contributions. We then set the planetary boundary at 3000 n-kg_*AE*_ per million US$, i.e. at three times the current emissions level. This might seem a rather permissive approach, but any boundary is more restrictive and useful than no boundary at all, and our pragmatic approach can very easily and quickly be adjusted as new scientific insights come along.

The equation below allows us to translate the EIO-LCA indicators into a boundary-compatible economic intensity for aerosol emissions, expressed in n-kg_*AE*_ per million US$:

$${\text{EIB}}\left[ {{\text{aerosol}}\;{\text{emissions}}} \right]=\mathop \sum \nolimits^{} \frac{{{\text{weighting}}\;{\text{factor}}\;\left( i \right)~ \times 1000}}{{{\text{EIOLCA}}\;{\text{average}}\;(i)}}$$

### Chemical pollution and environmental release of novel entities

As for aerosols, the planetary boundaries literature notes the existence of biophysical feedbacks and Earth system thresholds associated with chemical pollution. In addition to direct, but relatively local effects on ecosystems and human health, there are complex and wide-ranging systemic effects (Persson et al. 2013; Diamond et al. 2015). Chemically persistent, (bio)accumulative, systemically harmful synthetic substances should be created and used in closed systems, not applied or leaked into the environment, but the introduction of novel substances into the environment is still increasing and is now recognised as a major global concern (European Commission 2006; United Nations Environment Programme 2013). However, given the range of substances involved, the complexity of their physical and ecological interactions, and the limited access to information about industrially produced substances, no quantified planetary boundary has yet been proposed.

Because of the importance and pervasiveness of chemical pollution, and the unique role of business in controlling the global flows of synthetic chemical substances, we propose a tentative operational boundary. The approach and formula for calculating Economic intensity [Chemical Pollution] are the same as for aerosol emissions.

We selected appropriate emissions indicators from the EIO-LCA database, and weighted them according to simplifying assumptions about their environmental contribution. We convert these indicators into n-kg_*CP*_, a virtual chemical pollution unit constructed such that the average emissions intensities for the selected EIO-LCA indicators add up to 1000 n-kg_*CP*_ per million US$. We then set the planetary boundary arbitrarily at 3000 n-kg_*CP*_ per million US$, i.e. at three times the current emissions level. As for aerosols, this may be an overly generous boundary, but in the absence of scientific quantifications for a stronger constraint, we have opted to start with this flexible approach.

Table 4 shows the EIO-LCA indicators that we selected, together with their respective conversion factors to allow for combination into our investment boundary value.

**Table 4:** Translation of biophysical control variables for chemical pollution to economic intensity measures and investment boundaries

EIO-LCA indicator, *i*	Unit (per million US$)	Average value in EIO-LCA unit	Conversion factor	Average value in n-kg_*CP*_
Emissions				
Toxic releases into air	kg	223	0.56	125
Toxic releases into surface water	kg	33	3.8	125
Toxic releases into underground water	kg	56	2.2	125
Toxic releases into soil	kg	1,095	0.11	125
Impact criteria				
Ecotoxicity (high)	kg 1,4DCBe^a^	94	1.4	129
Ecotoxicity (low)	kg 1,4DCBe	88	1.4	121
Human health air quality	kg PM10e	3,451	0.024	83
Human health cancer (high)	kg benzene	1,331	0.054	72
Human health cancer (low)	kg benzene	199	0.054	11
Human health non-cancer (high)	kg toluene	1,963,657	4 × 10^− 5^	79
Human health non-cancer (low)	kg toluene	116,853	4 × 10^− 5^	5
Total	n-kg_***CP***_			1000

^a^The LCA characterization reference factor DCBe is dichlorobenzene equivalent

## Applying economic intensity boundaries to investment analysis

According to the financial service provider Bloomberg, currently about 70,000 stock-listed companies report their financials and their economic activities publicly, and could therefore be subject to an economic-intensity planetary boundaries analysis. Companies operating within the threshold economic intensities can be regarded to be operating within Earth’s ‘safe operating space’, while companies transgressing the boundaries are potentially accelerating the problematic trends of critical environmental processes. By extension, companies operating within the boundaries or that actively help other companies to do so, for instance by providing environmental products and services, are more likely to be able to navigate future resource constraints and also to benefit from stricter environmental regulations. Crucially for investors, these companies are more likely to prevail economically over their boundary-transgressing peers in a greener and more resource-constrained future.

However, from an investor’s perspective, a company-by-company analysis of the impact on the planetary boundaries is not tractable, so we propose a three-stage approach for applying the EIB framework. In a first step, planetary boundaries profiles can be established for clusters of companies. This blunt quantitative sub-industry clustering then needs adjustment to account for qualitative differences in environmental impacts. And finally, if asset management is to be an effective lever for sustainability, there is a need to identify both the ‘good’ environmentally friendly companies in the vanguard of changing a generally problematic sub-industry, and the ‘bad’ companies that fall short of expected environmental compatibility in their cluster.

### Sub-industry clustering

Industry classification schemes, such as the widely used Global Industry Classification Standard (GICS, MSCI, Inc. 2014), provide a taxonomy of sub-industries with global coverage of stock-listed companies. However, the EIO-LCA database uses industry sectors specified in the North American Industry Classification System (NAICS)^4^ as the basis for its detailed impact data (Carnegie Mellon University Green Design Institute 2016). Knowledge of the processes and activities of the NAICS sector categories and GICS sub-industries is needed in order to select and combine specific physical process-oriented indicators from the EIO-LCA database, to obtain the best possible representation of the overall impacts on emissions and resource consumption of company clusters. In other words, GICS sub-categories need to be recomposed in EIO-LCA terms. There is no one-size-fits-all scientific formula for this process—it relies on expert know-how about the typical activities of a company or of a GICS cluster of companies.

Once the EIO-LCA impacts have been obtained for the sub‑industry clusters, they can be rated against the economic intensity boundaries. At the sub-industry level, we propose using a logarithmic function to standardize the distance between the modelled impacts and the EIB value, for all boundaries. The nine EIB ratings can then be aggregated on an equally weighted basis to give an overall planetary boundaries performance rating. (Equal weighting may not be optimal, given that the planetary boundaries have such different properties and geospatial distributions (Häyhä et al. 2016), but we used it because as yet there is no scientific or operational consensus as to how to weigh boundaries against each other.) We can also accommodate effects of uncertainties by defining a range, say ± 20%, around this rating value. Uncertainties arise from the impact estimations, the simplifying assumptions we applied for the planetary boundaries, and the mapping of impacts from the EIO-LCA database to the GICS sub-industries. This ‘grey zone’ might be enlarged to account for additional uncertainties, for instance if an inhomogeneous group of companies has to be rated. In this way, sub-industries with a planetary boundaries impact rating above our uncertainty zone would be discarded outright, those in the uncertainty zone would pass the first cluster filter, and those below the grey-zone would be considered beneficial sub-industries from an environmental perspective, and also potentially interesting ones from an investor perspective.

In our initial analysis, this clustering approach brought our background universe of 70 000 stock-listed equities down to 16 environmentally active sub-industries, comprising a pool of approximately 2000–3000 investment candidates. We consider sub-industries to be ‘environmentally active’ when they have the potential to actively help other companies to reduce the stress on at least one of the planetary boundaries, for instance, through environmental technology, as opposed to merely having a low environmental footprint themselves.

### Cluster fine-tuning

The first reason for cluster adjustment is that the EIO-LCA’s life-cycle assessment is a ‘cradle-to-gate’ approach [termed scope 1 and 2 in LCA analysis, after the reporting categories of the widely used GHG Protocol standard (WRI and WBCSD 2004)]. It deals with upstream and in-house resource consumptions and emissions. It does not take into account the use and end-of-life phases of products and services. For many sub-industries, these phases make the greater contribution to their overall impact. For example, the CO_2_ and pollution emitted by an average car over its useful life is much greater than the direct emissions from its input materials and assembly.

A second reason is that the purely quantitative nature of EIO-LCA is unable to detect instances where qualitative aspects of a sub-industry’s activities mean that it should not be penalised for an apparently problematic in-house impact.

For example, there are many ways to use land. The extensive land use requirements for sustainable forestry will differ in important ways from the same land area used by a mining company. We argue that investor choices should reflect this difference. Another example is environmental services companies, which typically have high direct emissions in EIO-LCA (e.g., because they burn or land-fill waste). These emissions stem from ‘everyone else’s problem’—the companies are not generating emissions and waste on their own behalf; they are in the business of remediating existing environmental pollution created by other companies.

An effective investment model needs to be flexible enough to be able to account for impacts in the use-phase and end-of-life effects (scope 3, or ‘cradle to grave’), and also to factor in important qualitative differences in the in-house production phase.

These kinds of category adjustments reflect an emergent issue in LCA analysis, which has moved progressively through the ‘scopes’ from direct emissions/resource uses, to indirect emissions linked to consumption, and then to through-life impacts, and now even to ‘scope 4’ impacts. These involve quantifying environmental savings associated with specific ‘green’ products substituting mainstream ones (Frischknecht et al. 2016).

Although the starting point for our first-cut analysis weighted all the planetary boundaries equally in the overall rating, dealing with these two kinds of fine-tuning adjustment will require delving deeper into the EIB framework. This in turn will require careful scientific and technical justification for each case.

For example, in a forestry sub-industry, there are several possible dimensions of a scientific rationale for adjusting the investment model. For example, the extensive land use requirements are also large carbon sinks, affecting climate mitigation. The growing forests may also be protecting against soil erosion, attenuating changes in the water cycle, and providing important habitat for biodiversity.

### Individual company analysis

Obviously individual companies fall in a distribution around the average profile of their clusters. Identifying and discarding the unattractive companies in the ‘good’ sub-industries is a relatively straightforward process. Detecting attractive companies in ‘bad’ sub-industries is more challenging, as this depends on exogenous information (i.e., outside the GICS classification systems and EIO-LCA itself) about the company’s specific environmental performance. This information must be gathered from a wide range of networks and sources.

Financial asset managers can draw on existing investment expertise in environmentally themed equities, such as water, clean energy, and timber funds (Golden 2012; Bérubé et al. 2014). However, several aspects of the planetary boundaries framework do not align tightly with existing thematic funds, which means that there is still very little investment-oriented thematic research to draw upon.

An individual company analysis would therefore involve a detailed breakdown of the company’s products and services into the EIO-LCA activities (because these may deviate substantially from the sub-industry average). Just as for the sub-industry level assessment, individual adjustments are needed, at either the production level or at the level of the impact of the product or service with clients (scopes 3 and 4), to ensure that our investment universe really does include the strongest of green companies. Given the reputational risk of getting things wrong, the individual company analysis must also consider the full range of a company’s activities—some kinds of problematic products or services could compromise the positive impact of their environmental activities. This activity mix can then be used to compute and benchmark the environmental impact against the nine EIBs, giving a corporate planetary boundaries impact profile.

Promoting industry leadership for sustainability can be an important factor in the market positioning of investment products. This final filter step therefore needs to apply quite stringent criteria of exposure to the environmental theme. With the choices we made for our analysis, this reduced the investment universe by a further order of magnitude, to about 300 active ‘environmental contributors’.

## Recommendations, remaining challenges, conclusions

Using EIB for company analysis offers a new way of tracking the sustainability of companies in terms of both their core business activities and Earth’s non-negotiable biophysical constraints. Existing corporate environmental performance measures are often still relative, qualitative and subjective, dealing with what companies perceive to be their environmental burden (or their most pressing political issues).

It is still challenging to obtain reasonable approximations of the actual environmental impact of different economic activities and of the entire value chain of companies. However, that is what will ultimately determine whether a company’s business model, products and services will be among the winners or the losers in an environmentally challenging world. A technically robust assessment demands a much higher degree of transparency at all stages in the assessment: scientific knowledge about environmental changes, life-cycle impact analysis, and industry best practices. We have had to make many simplifying assumptions because actionable information is not available.

We suggest that in the context of the complex global environmental problems that the planetary boundaries framework highlights, the necessary process of information-sifting requires a radically greater level of dialogue between business and science communities (Frischknecht et al. 2016). Investors want to be responsive to the strategic importance of environmental products and services, the growing proportion of new environmental products, and the rising provision of environmental solutions. This capacity of companies to drive change for sustainability was an important motivation for us, as transdisciplinary professionals, in developing this framework.

The market positioning of investment products will play a decisive role in shifting behaviours to sustainable pathways. In our view, it is vital to distinguish companies that actively contribute to ‘bending the curves’ of environmental problems from those that passively have a low footprint because of the sectors they are in. Our approach allows us to make this distinction, and define a responsible investment universe with the balance shifted towards companies that make a positive difference to the world.

One major challenge underlies all of this: how can investors keep abreast of the rapidly evolving science of global environmental risks? We note that there is steady progress in deepening science-business dialogue, such as in recent events of the World Economic Forum (https://www.weforum.org/system-initiatives) and through new forums like Future Earth’s Knowledge Action Networks (http://futureearth.org/knowledge-action-networks). But we still need to make much more effort to align scientific and corporate language, timeframes of action and knowledge resources in ways that enable us to take steps towards sustainability together.

## Footnotes

1 Latest national data on many global economic and environmental indicators: http://databank.worldbank.org/data.

2 Global CO_2_ data: http://www.esrl.noaa.gov/gmd/ccgg/trends/global.html.

3 CML-IA database: http://www.universiteitleiden.nl/en/research/research-output/science/cml-ia-characterisation-factors.

4 The North American Industry Classification System (NAICS) is the standard used by US federal statistical agencies: http://www.census.gov/eos/www/naics.

## References

[CR1] Andersen SO, Halberstadt ML, Borgford-Parnell N (2013). Stratospheric ozone, global warming, and the principle of unintended consequences—an ongoing science and policy success story. J Air Waste Manag Assoc.

[CR2] Berkes F (2017). Environmental governance for the anthropocene? Social-ecological systems, resilience, and collaborative learning. Sustainability.

[CR3] Bérubé V, Ghal S, Tétrault J (2014). From indexes to insights: The rise of thematic investing. McKinsey Invest.

[CR4] Bouwman AF, Bierkens MF, Griffioen J (2013). Nutrient dynamics, transfer and retention along the aquatic continuum from land to ocean: towards integration of ecological and biogeochemical models. Biogeosciences.

[CR5] Busch T, Bauer R, Orlitzky M (2016). Sustainable development and financial markets old paths and new avenues. Bus Soc.

[CR6] Bush SR, Oosterveer P, Bailey M, Mol APJ (2015). Sustainability governance of chains and networks: a review and future outlook. J Clean Prod.

[CR7] Carnegie Mellon University Green Design Institute (2016) Assumptions, uncertainty, and other considerations with the EIO-LCA method. http://www.eiolca.net/Method/assumptions-and-uncertainty.html. Accessed 7 May 2018

[CR8] CBD (2014) Global Biodiversity Outlook 4: A mid-term assessment of progress towards the implementation of the Strat egic Plan for Biodiversity 2011–2020. Secretariat of the Convention on Biological Diversity, Montreal, Canada. pp 155. https://www.cbd.int/gbo4

[CR9] Ciais P, Stocker TF, Qin D, Plattner G-K, Tignor M, Allen SK, Boschung J, Nauels A, Xia Y, Bex V, Midgley PM (2013). Carbon and other biogeochemical cycles. Climate change 2013: the physical science basis. Contribution of Working Group I to the Fifth Assessment Report of the Intergovernmental Panel on Climate Change.

[CR10] Cole MJ, Bailey RM, New MG (2014). Tracking sustainable development with a national barometer for South Africa using a downscaled “safe and just space” framework. Proc Natl Acad Sci.

[CR11] Cooley SR, Doney SC (2009). Anticipating ocean acidification’s economic consequences for commercial fisheries. Environ Res Lett.

[CR12] Dahlmann F, Brammer S (2013). Corporate governance vs. corporate environmental governance: complementary or separate drivers of environmental performance?. Proc Int Assoc Bus Soc.

[CR13] Dao H, Peduzzi P, Chatenoux B (2015). Environmental limits and Swiss footprints based on Planetary Boundaries: a study commissioned by the Swiss Federal Office for the Environment (FOEN). UNEP/GRID-Geneva, Université de Genève.

[CR14] De Siqueira IR (2017). Development by trial and error: the authority of good enough numbers. Int Polit Soc.

[CR15] Dearing JA, Wang R, Zhang K (2014). Safe and just operating spaces for regional social-ecological systems. Glob Environ Change.

[CR16] Diamond ML, de Wit CA, Molander S (2015). Exploring the planetary boundary for chemical pollution. Environ Int.

[CR17] Doney SC, Mahowald N, Lima I (2007). Impact of anthropogenic atmospheric nitrogen and sulfur deposition on ocean acidification and the inorganic carbon system. Proc Natl Acad Sci.

[CR18] Edenhofer O, Pichs-Madruga R, Sokona Y et al Technical Summary. In: Edenhofer, O., R, Pichs-Madruga Y, Sokona E, Farahani S, Kadner K, Seyboth A, Adler I, Baum S, Brunner P, Eickemeier B, Kriemann J, Savolainen S, Schlömer C, von Stechow T, Zwickel, Minx JC (eds) Climate change 2014: mitigation of Climate Change. Contribution of Working Group III to the Fifth Assessment Report of the Intergovernmental Panel on Climate Change. Cambridge University Press, Cambridge, pp 33–107

[CR19] European Commission (2006) Regulation (EC) No 1907/2006 of the European Parliament and of the Council on the Registration, Evaluation, Authorisation and Restriction of Chemicals (REACH)

[CR20] FAO Forestry (2010). Global forest resources assessment.

[CR21] Feely RA, Doney SC, Cooley SR (2009). Ocean acidification: present conditions and future changes in a high-CO_2_ world. Oceanography.

[CR22] Fowler D, Coyle M, Skiba U et al (2013) The global nitrogen cycle in the twenty-first century. Philos Trans R Soc B Biol Sci 368: 10.1098/rstb.2013.0164 10.1098/rstb.2013.0164 PMC3682748 23713126

[CR23] Friedlingstein P, Gallego-Sala AV, Blyth EM, Cornell SE, Prentice IC, House JI, Downy CJ (2012). The Earth system feedbacks that matter for contemporary climate. Understanding the earth system: global change science for application.

[CR24] Frischknecht R, Stolz P, Tschümperlin L (2016). National environmental footprints and planetary boundaries: from methodology to policy implementation 59th LCA forum, Swiss Federal Institute of Technology, Zürich, June 12, 2015. Int J Life Cycle Assess.

[CR25] Galaz V, de Zeeuw A, Shiroyama H, Tripley D (2016). Planetary boundaries—governing emerging risks and opportunities. Solut J.

[CR26] Gattuso J-P, Magnan A, Billé R (2015). Contrasting futures for ocean and society from different anthropogenic CO2 emissions scenarios. Science.

[CR27] Gimenez C, Tachizawa EM (2012). Extending sustainability to suppliers: a systematic literature review. Supply Chain Manag Int J.

[CR28] Golden P (2012) Finding the future in thematic investing. Glob Invest 25–25

[CR29] Guinotte JM, Fabry VJ (2008). Ocean acidification and its potential effects on marine ecosystems. Ann N Y Acad Sci.

[CR30] Hahn R, Kuehnen M (2013). Determinants of sustainability reporting: a review of results, trends, theory, and opportunities in an expanding field of research. J Clean Prod.

[CR31] Hardt L (2013). The idea of good (enough) governance. the view from complexity economics. Argum Oecon.

[CR32] Häyhä T, Lucas PL, van Vuuren DP (2016). From planetary boundaries to national fair shares of the global safe operating space—How can the scales be bridged?. Glob Environ Change.

[CR33] Hoff H (2011). Understanding the nexus.

[CR34] Hofmann M, Schellnhuber H-J (2009). Oceanic acidification affects marine carbon pump and triggers extended marine oxygen holes. Proc Natl Acad Sci.

[CR35] IPCC (2014) Climate change 2014: synthesis report summary for policymakers. Contribution of Working Groups I, II and III to the Fifth Assessment Report of the Intergovernmental Panel on Climate Change

[CR36] IPCC (Intergovernmental Panel on Climate Change), UNEP, United Nations Environment Programme (2005). IPCC/TEAP Special Report on safeguarding the ozone layer and the global climate system: Issues related to hydrofluorocarbons and perfluorocarbons.

[CR37] Jarvis AJ, Leedal DT, Hewitt CN (2012). Climate-society feedbacks and the avoidance of dangerous climate change. Nat Clim Change.

[CR38] Kim I-N, Lee K, Gruber N (2014). Increasing anthropogenic nitrogen in the North Pacific Ocean. Science.

[CR39] Lambin EF, Meyfroidt P (2011). Global land use change, economic globalization, and the looming land scarcity. Proc Natl Acad Sci.

[CR40] Le Quéré C, Andrew RM, Canadell JG (2016). Global carbon budget 2016. Earth Syst Sci Data.

[CR41] Lindquist EJ, D’Annunzio R, Gerrand A (2012). Global forest land-use change 1990–2005.

[CR42] Linnenluecke MK, Birt J, Lyon J, Sidhu BK (2015). Planetary boundaries: implications for asset impairment. Acc Finance.

[CR43] MA (Millennium Ecosystem Assessment) (2005). Ecosystems and human well-being: synthesis.

[CR44] Mace GM, Masundire H, Baillie J (2005). Chap. 4: Biodiversity. Ecosystems and human well-being: current state and trends.

[CR45] Mace GM, Reyers B, Alkemade R (2014). Approaches to defining a planetary boundary for biodiversity. Glob Environ Change.

[CR46] MSCI, Inc (2014) Global industry classification standard (GICS®)

[CR47] Myhre G, Shindell D, Bréon FM, Stocker TF, Qin D, Plattner G-K, Tignor M, Allen SK, Boschung J, Nauels A, Xia Y, Bex V, Midgley PM (2013). Anthropogenic and natural radiative forcing. Climate change 2013: the physical science basis. Contribution of Working Group I to the Fifth Assessment Report of the Intergovernmental Panel on Climate Change.

[CR48] NOAA, NASA., UNEP (2011). Scientific assessment of ozone depletion: 2010. Executive summary.

[CR49] Nykvist B, Persson Å, Moberg F (2013). National environmental performance on planetary boundaries: a study for the Swedish Environmental Protection Agency.

[CR50] Pérez-Henriquez BL (2013) Environmental commodities markets and emissions trading: towards a low-carbon future. Routledge RFF Press, NY, USA, p 226. ISBN: 978-1617260940

[CR51] Persson LM, Breitholtz M, Cousins IT (2013). Confronting unknown planetary boundary threats from chemical pollution. Environ Sci Technol.

[CR52] Przychodzen J, Gómez-Bezares F, Przychodzen W, Larreina M (2016). ESG Issues among fund managers—factors and motives. Sustainability.

[CR53] Renneboog L, Ter Horst J, Zhang C (2008). The price of ethics and stakeholder governance: The performance of socially responsible mutual funds. Spec Issue Contract Corp Gov Eur Corp Gov Inst ECGI Symp Contract Corp Gov.

[CR54] Rockström J, Steffen W, Noone K, Persson Å, Chapin FS III, Lambin E, Lenton TM, Scheffer M, Folke C, Schellnhuber HJ, Nykvist B, De Wit C A, Hughes T, van der Leeuw S, Rodhe H, Sörlin S, Snyder PK, Costanza R, Svedin U, Falkenmark M, Karlberg L, Corell RW, Fabry VJ, Hansen J, Walker B, Liverman D, Richardson K, Crutzen P, Foley J (2009) Planetary boundaries: exploring the safe operating space for humanity. Ecol Soc 14 (Art. 32). https://www.ecologyandsociety.org/vol14/iss2/art32

[CR55] Saundry P (2016). Introduction (food-energy-water nexus special issue). J Environ Stud Sci.

[CR56] Schulz TF, Kypreos S, Barreto L, Wokaun A (2008). Intermediate steps towards the 2000 W society in Switzerland: an energy-economic scenario analysis. Energy Policy.

[CR57] Shiklomanov IA, Rodda JC (2003). World water resources at the beginning of the twenty-first century.

[CR58] Sparkes R, Cowton CJ (2004). The maturing of socially responsible investment: a review of the developing link with corporate social responsibility. J Bus Ethics.

[CR59] Steffen W, Richardson K, Rockstrom J (2015). Planetary boundaries: guiding human development on a changing planet. Science.

[CR60] Sutton MA, Bleeker A, Howard CM (2013). Our Nutrient World: The challenge to produce more food and energy with less pollution.

[CR61] Underdal A (2010). Complexity and challenges of long-term environmental governance. Glob Environ Change-Hum Policy Dimens.

[CR62] UNFCCC (2015) Adoption of the Paris Agreement. Conference of the Parties, Agenda item 4(b), 21st session, Paris, 30 November to 11 December 2015

[CR63] United Nations Environment Programme (2013). Global chemicals outlook: towards sound management of chemicals.

[CR64] Vatn A (2015). Markets in environmental governance. From theory to practice. Ecol Econ.

[CR65] WRI and WBCSD (2004). The greenhouse gas protocol: a corporate accounting and reporting standard.

